# Association between smoking cessation duration and prediabetes among former smokers: A secondary dataset analysis of the Korea National Health and Nutrition Examination Survey 2015–2024

**DOI:** 10.18332/tid/236889

**Published:** 2026-10-07

**Authors:** Minchai Kim, Hongji Lee, Yeon Woo Oh, Eun-Cheol Park

**Affiliations:** 1 Premedical Courses, Yonsei University College of Medicine, Seoul, Republic of Korea; 2 Department of Biostatistics and Computing, Yonsei University Graduate School, Seoul, Republic of Korea; 3 Department of Preventive Medicine, Yonsei University College of Medicine, Seoul, Republic of Korea; 4 Institute of Health Services Research, Yonsei University, Seoul, Republic of Korea

**Keywords:** smoking cessation, prediabetic state, ex-smokers, sex factors, Republic of Korea

## Abstract

**INTRODUCTION:**

Prediabetes is an early stage of glucose dysregulation and an important target for diabetes prevention. Smoking is a modifiable risk factor for impaired glucose metabolism, and metabolic risk after smoking cessation may differ by time since cessation. This study examined the association between smoking cessation duration and prediabetes among Korean adult former smokers.

**METHODS:**

This secondary dataset analysis used data from the Korea National Health and Nutrition Examination Survey (KNHANES), a nationwide cross-sectional survey conducted in the Republic of Korea, from 2015 to 2024, and included 8790 former smokers aged ≥30 years without diabetes. Smoking cessation duration was the primary exposure, and prediabetes was defined as fasting plasma glucose 100–125 mg/dL or hemoglobin A1c 5.7–6.4%. Sex-stratified multivariable logistic regression models were used to estimate adjusted odds ratios (AORs) and 95% confidence intervals (CIs).

**RESULTS:**

Prediabetes was present in 4205 men (56.9%) and 478 women (34.3%). Among men, each additional year since smoking cessation was associated with lower odds of prediabetes (AOR=0.99; 95% CI: 0.99–1.00; p=0.019), whereas no significant association was observed among women (AOR=1.00; 95% CI: 0.99–1.02; p=0.554). In subgroup analyses among men, inverse associations were observed in those with 10–19 pack-years (AOR=0.99; 95% CI: 0.98–1.00) and ≥20 pack-years (AOR=0.99; 95% CI: 0.98–1.00).

**CONCLUSIONS:**

Longer smoking cessation duration was associated with lower odds of prediabetes among Korean male former smokers, whereas no comparable association was observed among women. The association was more evident in men with greater cumulative smoking exposure. Because of the cross-sectional design, these findings should be interpreted as associations and do not establish a causal effect of smoking cessation duration on metabolic risk.

## INTRODUCTION

Diabetes is a major chronic disease that contributes substantially to morbidity, mortality, and healthcare burden worldwide^1^. The International Diabetes Federation estimated that 589 million adults aged 20–79 years were living with diabetes in 2024, and projected that this number will increase to 853 million by 2050^1^. Type 2 diabetes accounts for more than 90% of all diabetes cases and is closely associated with modifiable lifestyle-related factors, including obesity, physical inactivity, unhealthy diet, and smoking^1,2^. Targeting these risk factors through lifestyle modification remains central to diabetes prevention^2,3^.

Smoking is an important modifiable factor related to glucose metabolism. Previous studies have suggested that smoking may contribute to diabetes through oxidative stress, chronic inflammation, insulin resistance, and pancreatic beta-cell dysfunction^4,5^. Several epidemiological studies have reported that current smokers have a higher risk of type 2 diabetes than never smokers, with evidence of a dose-response relationship according to smoking intensity^6^. However, findings on smoking cessation have been less straightforward. Some studies have reported a temporary increase in diabetes risk after cessation, partly due to post-cessation weight gain^7,8^. Others have shown that diabetes risk decreases with longer periods of abstinence from smoking^9^. These findings suggest that metabolic risk among former smokers may differ according to the duration of smoking cessation.

At the same time, the potential for reverse causality should be considered when evaluating the relationship between smoking cessation and diabetes risk^10,11^. Individuals diagnosed with diabetes may change their lifestyles, including smoking behavior, diet, and physical activity following diagnosis. By contrast, prediabetes, an earlier stage of glucose dysregulation before overt diabetes, is less likely to prompt such behavioral changes. Furthermore, prediabetes serves as a strong predictor of future diabetes, as a substantial proportion of individuals with prediabetes eventually progress to diabetes^12^. For these reasons, prediabetes may serve as a useful proxy for assessing early metabolic risk associated with smoking cessation, while reducing the potential for reverse causality.

This study aimed to examine the association between smoking cessation duration and prediabetes among Korean adult former smokers using data from the Korea National Health and Nutrition Examination Survey. By examining time since cessation, this study aimed to clarify whether prediabetes risk differs according to the length of time after quitting, an issue suggested by prior studies showing that diabetes risk changes over time after smoking cessation^7-9^.

## METHODS

### Study design

This study was a secondary dataset analysis of the Korea National Health and Nutrition Examination Survey (KNHANES), a nationwide cross-sectional survey conducted in the Republic of Korea, using data collected from 2015 to 2024. Eligible participants were former smokers aged ≥30 years without diabetes. Participants aged <30 years, never smokers, current smokers, participants with diabetes, and those with missing values for smoking cessation duration, prediabetes status, or relevant covariates were excluded. The final analytic sample included 8790 former smokers (7396 men and 1394 women).

### Data source

KNHANES is an ongoing nationwide survey administered by the Korea Disease Control and Prevention Agency (KDCA). It consists of health interviews, health examinations, and nutrition surveys designed to evaluate the health status, health-related behaviors, and nutritional intake of the Korean population. Using a complex, multistage probability sampling design, KNHANES provides nationally representative data on the non-institutionalized Korean population^13^. Considering potential sex differences in smoking behavior, body composition, and metabolic risk profiles, analyses were conducted separately for men and women^14,15^.

*Exposure (smoking cessation duration)*

Former smoker status was determined based on self-reported smoking history, and smoking cessation duration was calculated as the number of years elapsed since smoking cessation.

In the primary analysis, smoking cessation duration was treated as a continuous variable to estimate the change in the odds of prediabetes for each 1-year increase in cessation duration. Additionally, smoking cessation duration was categorized into 4-year intervals: 0–3, 4–7, 8–11, 12–15, 16–19, 20–23, 24–27, 28–31, 32–35, and ≥36 years. Using the interval of 0–3 years as the reference category, we evaluated the pattern of association between smoking cessation duration and prediabetes across different post-cessation periods.

*Outcome (prediabetes)*

The outcome variable was prediabetes status. Individuals with diabetes – defined as a self-reported physician diagnosis of diabetes, fasting plasma glucose (FPG) ≥126 mg/dL, or hemoglobin A1c (HbA1c) ≥6.5% – were excluded prior to analysis. Among the remaining participants, prediabetes was defined as FPG of 100–125 mg/dL or HbA1c of 5.7–6.4%, while those meeting neither criterion were classified as having normoglycemia.

*Covariates*

Covariates included age group, body mass index (BMI), pack-years, age at smoking initiation, household income, employment status, marital status, alcohol consumption, hypertension, and aerobic physical activity. Age group was categorized as 30–39, 40–49, 50–59, 60–69, and ≥70 years. BMI (kg/m^2^) was categorized as: <23 (including underweight participants), 23–24.9, and ≥25; participants with BMI <18.5 were therefore included in the <23 group rather than analyzed separately. Age at smoking initiation was categorized as 0–19, 20–24, and ≥25 years in the multivariable models and subgroup analyses; these broader age bands were retained to maintain adequate numbers within each sex-specific stratum. Household income was categorized into quartiles. Employment status was classified as employed or unemployed, and marital status was classified as ever married or never married. Alcohol consumption was categorized as 0–4 versus ≥5 drinks per sitting. In the KNHANES questionnaire, the amount consumed on one occasion is counted in glasses regardless of alcohol type; one 355 mL can of beer is counted as 1.5 beer glasses. Hypertension was defined as systolic blood pressure ≥140 mmHg, diastolic blood pressure ≥90 mmHg, or current use of antihypertensive medication. KNHANES classifies vigorous-intensity activity as activity performed for at least 10 minutes that causes marked breathlessness or a very rapid heart rate, and moderate-intensity activity as activity performed for at least 10 minutes that causes slight breathlessness or a slightly faster heart rate. Participants were considered to meet the aerobic physical activity recommendation if they reported ≥150 min/week of moderate-intensity activity, ≥75 min/week of vigorous-intensity activity, or an equivalent combination, with 1 minute of vigorous activity counted as 2 minutes of moderate activity.

### Statistical analysis

Multivariable logistic regression models were used to examine the association between smoking cessation duration and prediabetes. The models were adjusted for age group, BMI, pack-years, age at smoking initiation, household income, employment status, marital status, alcohol consumption, aerobic physical activity, and hypertension. Given substantial sex differences in smoking-related behaviors and metabolic characteristics, all analyses were performed separately for men and women^14,15^. In the main analysis, smoking cessation duration was included as a continuous variable to estimate the change in odds of prediabetes per additional year since cessation. Additionally, smoking cessation duration was grouped into 4-year intervals to examine the pattern across cessation-duration groups.

Subgroup analyses were conducted to examine whether the association between smoking cessation duration and prediabetes differed according to smoking-related characteristics. These analyses were stratified by pack-years, age at smoking initiation, and age at smoking cessation.

Sensitivity analyses were conducted using three alternative definitions of prediabetes: FPG-defined prediabetes (FPG 100–125 mg/dL), HbA1c-defined prediabetes (HbA1c 5.7–6.4%), and prediabetes defined as meeting both criteria.

All statistical tests were two-sided, and statistical significance was defined as p<0.05. All analyses were performed using R software (version 4.5.3; R Foundation for Statistical Computing, Vienna, Austria)^16^.

## RESULTS

Prediabetes was present in 4205 men (56.9%) and 478 women (34.3%). In both sexes, participants with prediabetes were older and had less favorable metabolic profiles than participants with normoglycemia. Among men, the proportion of participants aged ≥50 years was higher in the prediabetes group than in the normoglycemia group (75.1% vs 59.3%), as were the proportions with BMI of ≥25 kg/m^2^ (49.1% vs 35.1%) and hypertension (36.5% vs 22.1%). Similar patterns were observed among women: participants with prediabetes were more frequently aged ≥50 years (56.0% vs 23.5%), had body mass index of ≥25 kg/m^2^ (44.8% vs 20.7%), and had hypertension (23.8% vs 8.3%) compared with women with normoglycemia. The mean cumulative smoking exposure was also higher in participants with prediabetes than in those with normoglycemia in both men (19.9 vs 15.3 pack-years) and women (6.2 vs 3.5 pack-years) (Table 1).

**Table 1:** Baseline characteristics of former smokers aged ≥30 years without diabetes, by sex and prediabetes status, a secondary dataset analysis of the Korea National Health and Nutrition Examination Survey (KNHANES), Republic of Korea, 2015–2024 (N=8790)

*Characteristics*	*Men*			*Women*		
	*Total*	*Normal*	*Prediabetes^a^*	*Total*	*Normal*	*Prediabetes*
**Total**	7396 (100)	3191 (43.1)	4205 (56.9)	1394 (100)	916 (65.7)	478 (34.3)
**Age** (years)						
30–39	973 (13.2)	619 (19.4)	354 (8.4)	482 (34.6)	389 (42.5)	93 (19.5)
40–49	1371 (18.5)	682 (21.4)	689 (16.4)	429 (30.8)	312 (34.1)	117 (24.5)
50–59	1588 (21.5)	644 (20.2)	944 (22.4)	227 (16.3)	121 (13.2)	106 (22.2)
60–69	1776 (24.0)	647 (20.3)	1129 (26.8)	143 (10.3)	62 (6.8)	81 (16.9)
≥70	1688 (22.8)	599 (18.8)	1089 (25.9)	113 (8.1)	32 (3.5)	81 (16.9)
**Smoking cessation duration** (years)	15.6 ± 12.5	15.2 ± 12.2	15.9 ± 12.6	13.1 ± 10.1	12.1 ± 9.3	14.8 ± 11.4
**Pack-years**	17.9 ± 18.0	15.3 ± 16.8	19.9 ± 18.6	4.4 ± 8.5	3.5 ± 6.5	6.2 ± 11.3
**Smoking initiation age** (years)						
0–19	3866 (52.3)	1739 (54.5)	2127 (50.6)	509 (36.5)	377 (41.2)	132 (27.6)
20–24	2838 (38.4)	1191 (37.3)	1647 (39.2)	524 (37.6)	354 (38.6)	170 (35.6)
≥25	692 (9.4)	261 (8.2)	431 (10.2)	361 (25.9)	185 (20.2)	176 (36.8)
**Smoking quit age** (years)						
0–29	1830 (24.7)	982 (30.8)	848 (20.2)	795 (57.0)	601 (65.6)	194 (40.6)
30–39	2143 (29.0)	1003 (31.4)	1140 (27.1)	291 (20.9)	191 (20.9)	100 (20.9)
40–49	1678 (22.7)	626 (19.6)	1052 (25.0)	154 (11.0)	70 (7.6)	84 (17.6)
≥50	1745 (23.6)	580 (18.2)	1165 (27.7)	154 (11.0)	54 (5.9)	100 (20.9)
**Body mass index** (kg/m²)						
<23	2108 (28.5)	1114 (34.9)	994 (23.6)	713 (51.1)	555 (60.6)	158 (33.1)
23–24.9	2103 (28.4)	956 (30.0)	1147 (27.3)	277 (19.9)	171 (18.7)	106 (22.2)
≥25	3185 (43.1)	1121 (35.1)	2064 (49.1)	404 (29.0)	190 (20.7)	214 (44.8)
**Income**						
Low	1161 (15.7)	454 (14.2)	707 (16.8)	222 (15.9)	110 (12.0)	112 (23.4)
Mid-Low	1754 (23.7)	734 (23.0)	1020 (24.3)	374 (26.8)	247 (27.0)	127 (26.6)
Mid-High	2008 (27.1)	879 (27.5)	1129 (26.8)	436 (31.3)	300 (32.8)	136 (28.5)
High	2473 (33.4)	1124 (35.2)	1349 (32.1)	362 (26.0)	259 (28.3)	103 (21.5)
**Employment status**						
Unemployed	1965 (26.6)	768 (24.1)	1197 (28.5)	769 (55.2)	533 (58.2)	236 (49.4)
Employed	5431 (73.4)	2423 (75.9)	3008 (71.5)	625 (44.8)	383 (41.8)	242 (50.6)
**Marital status**						
Ever married	6863 (92.8)	2863 (89.7)	4000 (95.1)	1228 (88.1)	780 (85.2)	448 (93.7)
Never married	533 (7.2)	328 (10.3)	205 (4.9)	166 (11.9)	136 (14.8)	30 (6.3)
**Alcohol consumption**						
0–4 drinks/sitting	4032 (54.5)	1747 (54.7)	2285 (54.3)	951 (68.2)	622 (67.9)	329 (68.8)
≥5 drinks/sitting	3364 (45.5)	1444 (45.3)	1920 (45.7)	443 (31.8)	294 (32.1)	149 (31.2)
**Aerobic physical activity**						
No	3965 (53.6)	1599 (50.1)	2366 (56.3)	838 (60.1)	533 (58.2)	305 (63.8)
Yes	3431 (46.4)	1592 (49.9)	1839 (43.7)	556 (39.9)	383 (41.8)	173 (36.2)
**Hypertension**						
No	5156 (69.7)	2487 (77.9)	2669 (63.5)	1204 (86.4)	840 (91.7)	364 (76.2)
Yes	2240 (30.3)	704 (22.1)	1536 (36.5)	190 (13.6)	76 (8.3)	114 (23.8)

Continuous variables are presented as mean ± SD; categorical variables are presented as n (%). SD: standard deviation.

a Prediabetes was defined as a fasting plasma glucose level of 100–125 mg/dL or a glycated hemoglobin (HbA1c) level of 5.7–6.4%.

In multivariable logistic regression, longer smoking cessation duration was associated with lower odds of prediabetes among men. Each additional year since smoking cessation was associated with lower odds of prediabetes in men (adjusted odds ratio, AOR=0.99; 95% CI: 0.99–1.00; p=0.019). In contrast, no statistically significant association was observed among women (AOR=1.00; 95% CI: 0.99–1.02; p=0.554) (Table 2).

**Table 2:** Adjusted association between smoking cessation duration and prediabetes among former smokers aged ≥30 years without diabetes, a secondary dataset analysis of KNHANES, Republic of Korea, 2015–2024 (men N=7396; women N=1394)

*Variables*	*Men*		*Women*	
	*AOR (95% CI)*	*p*	*AOR (95% CI)*	*p*
**Smoking cessation duration** (years)	0.99 (0.99–1.00)	0.019	1.00 (0.99–1.02)	0.554
**Age** (years)				
30–39 (ref.)	1.00		1.00	
40–49	1.62 (1.36–1.94)	<0.001	1.38 (0.98–1.95)	0.070
50–59	2.28 (1.88–2.76)	<0.001	3.22 (2.09–4.94)	<0.001
60–69	3.21 (2.47–4.17)	<0.001	4.68 (2.72–8.05)	<0.001
≥70	3.48 (2.74–4.42)	<0.001	7.78 (3.70–16.41)	<0.001
**Pack-years**	1.01 (1.00–1.01)	0.006	1.01 (1.00–1.01)	0.006
**Smoking initiation age** (years)				
0–19 (ref.)	1.00		1.00	
20–24	1.10 (0.99–1.22)	0.069	1.07 (0.79–1.45)	0.665
≥25	1.16 (0.97–1.40)	0.103	0.89 (0.59–1.32)	0.551
**Body mass index** (kg/m²)				
<23 (ref.)	1.00		1.00	
23–24.9	1.38 (1.21–1.56)	<0.001	1.87 (1.36–2.58)	<0.001
≥25	2.22 (1.96–2.50)	<0.001	3.75 (2.81–5.00)	<0.001
**Income group**				
Low (ref.)	1.00		1.00	
Mid-Low	1.09 (0.92–1.28)	0.322	0.98 (0.65–1.47)	0.918
Mid-High	1.14 (0.96–1.35)	0.139	0.98 (0.65–1.49)	0.940
High	1.09 (0.92–1.30)	0.328	0.92 (0.60–1.41)	0.705
**Employment status**				
Employed (ref.)	1.00		1.00	
Unemployed	0.93 (0.81–1.06)	0.249	1.12 (0.87–1.46)	0.373
**Marital status**				
Ever married (ref.)	1.00		1.00	
Never married	0.79 (0.64–0.97)	0.022	0.58 (0.37–0.91)	0.018
**Alcohol consumption**				
0–4 drinks/sitting (ref.)	1.00		1.00	
≥5 drinks/sitting	1.11 (1.00–1.23)	0.053	1.03 (0.78–1.35)	0.835
**Aerobic physical activity**				
No (ref.)	1.00		1.00	
Yes	0.84 (0.77–0.93)	0.001	0.98 (0.76–1.26)	0.848
**Hypertension**				
No (ref.)	1.00		1.00	
Yes	1.43 (1.28–1.61)	<0.001	1.06 (0.72–1.58)	0.767

AOR: adjusted odds ratio. CI: confidence interval.

In the 4-year grouped analysis among men, the estimates generally decreased with longer smoking cessation duration. Compared with the reference group of 0–3 years, AORs were approximately 0.91 for cessation durations of 4–15 years and ranged from 0.74 to 0.80 for cessation durations of ≥16 years, indicating a more pronounced inverse pattern with longer cessation duration (Figure 1). Among women, estimates were more variable with wider confidence intervals, and no consistent inverse pattern was observed across cessation-duration categories (Figure 2).

**Figure 1:**
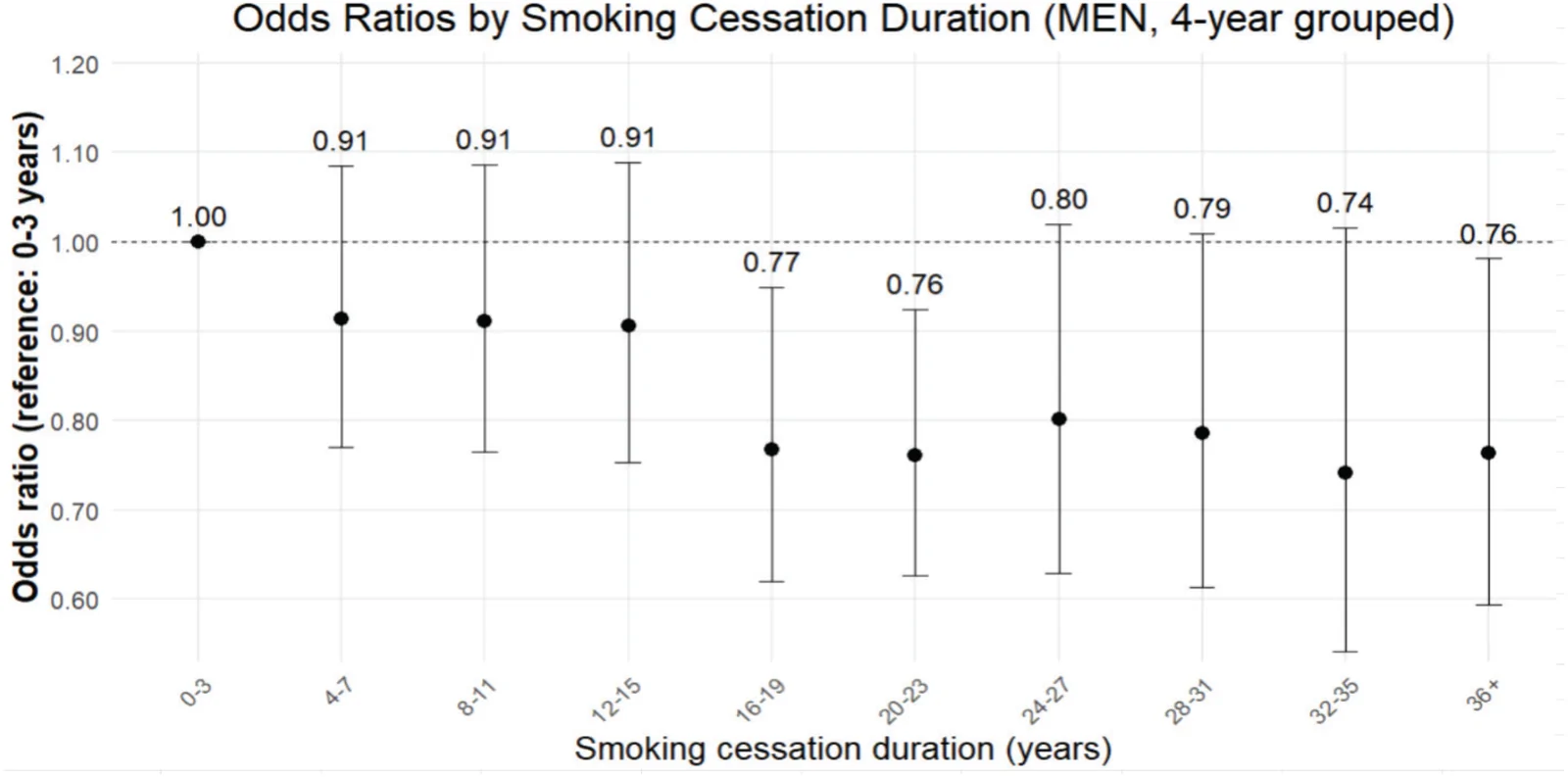
Adjusted odds ratios for prediabetes according to smoking cessation duration among male former smokers aged ≥30 years without diabetes, a secondary dataset analysis of KNHANES, Republic of Korea, 2015–2024 (N=7396)

**Figure 2:**
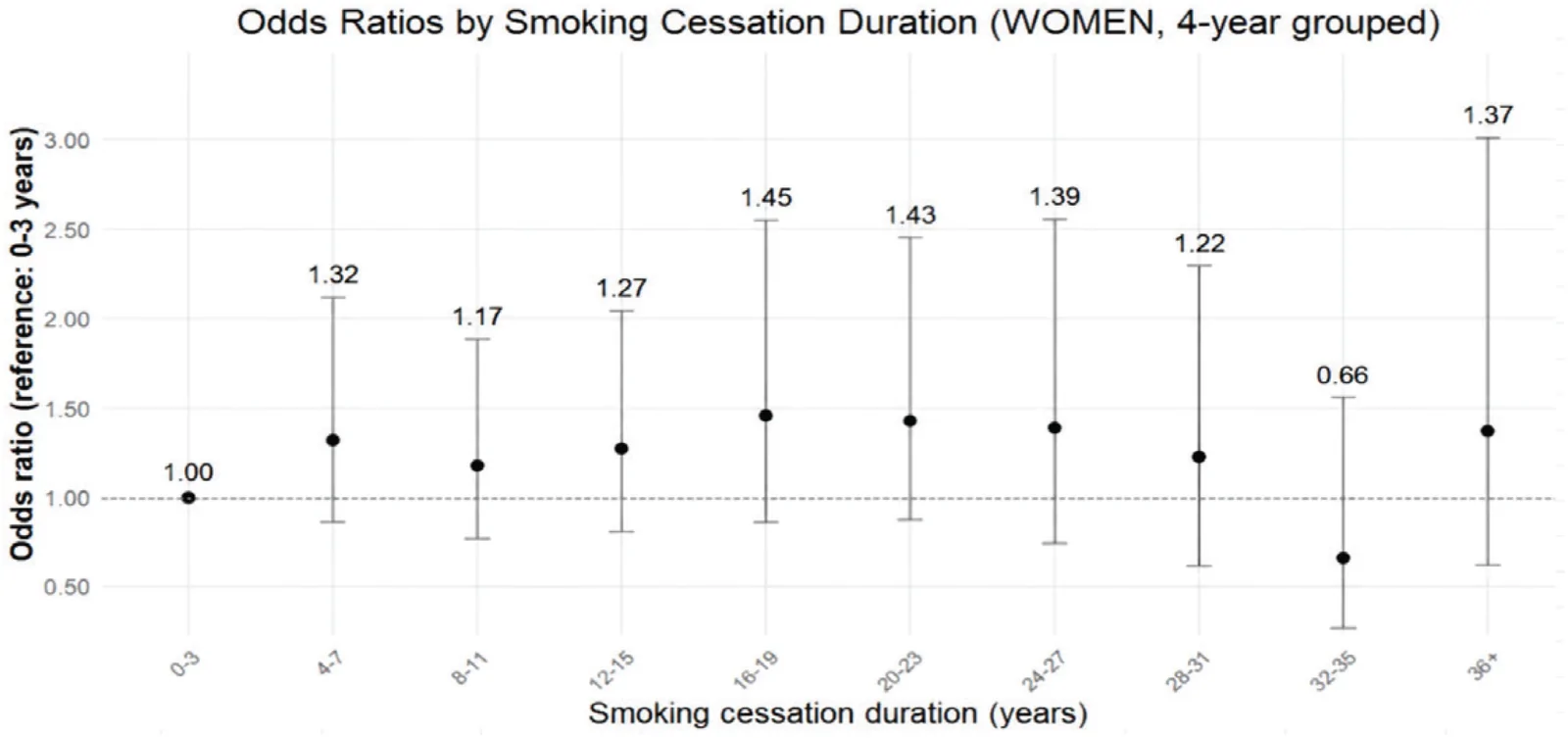
Adjusted odds ratios for prediabetes according to smoking cessation duration among female former smokers aged ≥30 years without diabetes, a secondary dataset analysis of KNHANES, Republic of Korea, 2015–2024 (N=1394)

In subgroup analyses involving smoking-related variables, the inverse association between smoking cessation duration and prediabetes among men was most evident among participants with greater cumulative smoking exposure (Table 3). Specifically, longer cessation duration was associated with lower odds of prediabetes among men with 10–19 pack-years (AOR=0.99; 95% CI: 0.98–1.00; p=0.026) and ≥20 pack-years (AOR=0.99; 95% CI: 0.98–1.00; p=0.008). Inverse associations were also observed among men who initiated smoking at ages 20–24 years (AOR= 0.99; 95% CI: 0.98–1.00; p=0.028) and those who quit at ages 40–49 years (AOR=0.98; 95% CI: 0.96–1.00; p=0.016). Corresponding associations among women were not statistically significant (all p≥0.05). Non-smoking-related subgroup analyses are provided in Supplementary file Table 1.

**Table 3:** Adjusted association between smoking cessation duration and prediabetes, by smoking-related subgroups, among former smokers aged ≥30 years without diabetes, a secondary dataset analysis of KNHANES, Republic of Korea, 2015–2024 (men N=7396; women N=1394)

*Subgroup*	*Category*	*Men*		*Women*	
		*AOR (95% CI)*	*p*	*AOR (95% CI)*	*p*
**Pack-years**					
	0–9	1.01 (1.00–1.01)	0.172	1.01 (0.99–1.02)	0.363
	10–19	0.99 (0.98–1.00)	0.026	0.97 (0.91–1.04)	0.344
	≥20	0.99 (0.98–1.00)	0.008	1.05 (0.94–1.16)	0.392
**Smoking initiation age** (years)					
	0–19	0.99 (0.99–1.00)	0.112	1.03 (1.00–1.06)	0.083
	20–24	0.99 (0.98–1.00)	0.028	0.99 (0.97–1.02)	0.549
	≥25	1.01 (0.99–1.02)	0.586	1.01 (0.98–1.03)	0.659
**Smoking quit age** (years)					
	0–29	1.00 (0.99–1.01)	0.821	1.02 (1.00–1.05)	0.099
	30–39	1.00 (0.99–1.02)	0.923	1.05 (1.00–1.10)	0.053
	40–49	0.98 (0.96–1.00)	0.016	1.04 (0.97–1.12)	0.316
	≥50	1.00 (0.98–1.01)	0.493	1.00 (0.95–1.06)	0.951

AOR: adjusted odds ratio. CI: confidence interval.

Sensitivity analyses using alternative definitions of prediabetes generally supported the sex-specific pattern observed in the main analysis. Among men, the association was not statistically significant when prediabetes was defined using FPG criteria only (AOR=1.00; 95% CI: 0.99–1.00; p=0.414), whereas inverse associations were observed using HbA1c criteria only (AOR=0.99; 95% CI: 0.99–1.00; p=0.001) and when both FPG and HbA1c criteria were met (AOR=0.99; 95% CI: 0.99–1.00; p=0.041). Among women, no statistically significant association was observed under any diagnostic definition (all p≥0.05) (Table 4).

**Table 4:** Sensitivity analyses of smoking cessation duration and prediabetes among former smokers aged ≥30 years without diabetes, a secondary dataset analysis of KNHANES, Republic of Korea, 2015–2024 (men N=7396; women N=1394)

*Diagnostic criteria*	*Men*		*Women*	
	*AOR (95% CI)*	*p*	*AOR (95% CI)*	*p*
FPG only^a^	1.00 (0.99–1.00)	0.414	1.01 (0.99–1.02)	0.315
HbA1c only^b^	0.99 (0.99–1.00)	0.001	1.01 (0.99–1.02)	0.302
Both FPG and HbA1c^c^	0.99 (0.99–1.00)	0.041	1.02 (1.00–1.04)	0.084

AOR: adjusted odds ratio. CI: confidence interval. FPG: fasting plasma glucose. HbA1c: glycated hemoglobin.

a Among individuals without diabetes, prediabetes was defined as a fasting plasma glucose level of 100–125 mg/dL.

b Among individuals without diabetes, prediabetes was defined as an HbA1c level of 5.7–6.4%.

c Among individuals without diabetes, prediabetes was defined as a fasting plasma glucose level of 100–125 mg/dL and an HbA1c level of 5.7–6.4%.

## DISCUSSION

In this study of Korean adult former smokers without diabetes, longer smoking cessation duration was associated with lower odds of prediabetes among men, whereas no comparable association was observed among women. This pattern persisted after adjustment for demographic, socioeconomic, lifestyle-related, and clinical covariates. The 4-year grouped analysis also showed a generally stronger inverse pattern at longer cessation durations, particularly from approximately 16 years after cessation. Given the cross-sectional design, these findings describe an association and should not be interpreted as evidence that a specific duration of abstinence causes a reduction in prediabetes risk.

The direction of the main finding is biologically plausible. Cigarette smoking has been linked to insulin resistance, systemic inflammation, oxidative stress, abdominal adiposity, and impaired glucose homeostasis^4,6,7,17,18^. A longer interval after cessation may therefore reflect more prolonged recovery from smoking-related metabolic burden, particularly among individuals with substantial prior exposure. This is consistent with cohort studies showing that the metabolic consequences of cessation are dynamic: diabetes risk may be higher in the early years after quitting and then weaken over time as the long-term effects of abstinence become more apparent^7,8,19^.

The subgroup analyses further support the importance of cumulative smoking burden. Among men, the inverse association was more evident in participants with ≥10 pack-years, indicating that smoking burden may be an important context for interpreting the association between cessation duration and early glycemic risk. Heavier cumulative smoking exposure has been associated with metabolic disruption through pathways involving insulin resistance, inflammation, oxidative stress, and adiposity^4,17,18^. Thus, the observed association between cessation duration and glycemic status may be more apparent among individuals with greater accumulated smoking-related exposure; however, this interpretation remains observational.

The absence of a significant inverse association among women should be interpreted mainly in light of several factors. First, the number of female former smokers was much smaller than the number of male former smokers, and their cumulative smoking exposure was also lower, which likely widened confidence intervals and limited the ability to detect an association. In addition, cumulative smoking exposure was substantially lower among women, which may have made the relationship between cessation duration and smoking-related metabolic burden less apparent. Furthermore, social under-reporting of smoking among Korean women may have further reduced the accuracy of smoking-history classification^20-22^. Lastly, sex-specific metabolic factors may also contribute. Menopausal transition, changes in fat distribution, and differences in insulin sensitivity can modify cardiometabolic risk^15,23-26^, so the metabolic consequences of smoking cessation among women may be more strongly intertwined with menopause-related adiposity and weight change.

The stronger finding for HbA1c-defined prediabetes than for fasting plasma glucose-defined prediabetes may reflect differences in what these biomarkers capture. Fasting plasma glucose represents glucose concentration at a single fasting time point and is influenced by short-term factors such as recent diet, fasting duration, hepatic glucose output, sleep, and acute stress. By contrast, HbA1c reflects average glycemic exposure over the preceding several weeks and may therefore be more sensitive to sustained, low-grade metabolic dysregulation related to insulin resistance, inflammation, and adiposity^27-29^. Prior studies have shown meaningful discordance between HbA1c-based and fasting glucose-based classification of diabetes and prediabetes, including in Korean populations^29,30^. In the present study, the association with cessation duration appearing for HbA1c but not for fasting glucose, suggests that longer abstinence may be more closely related to chronic glycemic burden than to isolated fasting glycemia. This interpretation should remain cautious, however, because HbA1c can also be affected by erythrocyte turnover and other non-glycemic factors^27,31^.

### Limitations

This study has several limitations. First, because the analysis was cross-sectional, temporal order and causality cannot be established. Longer cessation duration may be associated with lower prediabetes odds, but this design cannot determine whether cessation duration itself reduced risk. Second, smoking-related variables, including cessation duration, smoking initiation age, and smoking amount, were based on self-report and may be affected by recall error or social desirability bias. This issue is especially important for women in Korea, among whom smoking history may be under-reported^20-22^. Third, residual confounding remains possible despite adjustment for demographic, socioeconomic, behavioral, and clinical factors. Alcohol consumption was also self-reported and may be affected by recall or reporting error. Diet, family history of diabetes, medication use, weight change after smoking cessation, menopausal status, and detailed physical activity were not fully accounted for. Future prospective studies should follow former smokers from the time of cessation and repeatedly assess FPG, HbA1c, body weight, and body composition. Incorporating objective smoking biomarkers and sex-specific factors, including menopausal transition and post-cessation changes in adiposity, may also help reduce exposure misclassification and clarify differences between men and women.

## CONCLUSIONS

Longer smoking cessation duration was associated with lower odds of prediabetes among Korean male former smokers, whereas no comparable association was observed among female former smokers. The association was more evident among men with greater cumulative smoking exposure. These findings support a sex-specific association between time since smoking cessation and prediabetes in former smokers, but prospective studies are needed to establish temporality and causality.

## Supplementary Material



## Data Availability

The data supporting this research are available from the following link: https://knhanes.kdca.go.kr/knhanes/main.do
