# Supplementary material for: Association between smoking cessation duration and prediabetes among former smokers: A secondary dataset analysis of the Korea National Health and Nutrition Examination Survey 2015–2024
Source: Tob Induc Dis. 2026 Oct 7;24:10.18332/tid/236889. doi: 10.18332/tid/236889 (PMC13648281; doi:10.18332/tid/236889)
Supplement: Supplementary file 1 [file TID-24-186-s1.pdf]

Tobacco Induced Diseases

Supplementary file

© 2026 Kim M. et al.

DOI:
10.18332/tid/236889

The content has been provided by the author(s) and has not been reviewed, verified, or endorsed by European Publishing. It may not have undergone peer review. The views, opinions, and recommendations expressed are solely those of the author(s) and do not necessarily reflect the position of European Publishing. European Publishing accepts no responsibility or liability for any consequences arising from the use of, or reliance on, this content.

Supplementary Table 1. Association between smoking cessation duration and prediabetes by non-smoking-related subgroups

Subgroup	Category	Men<br>aOR (95% CI)	Women<br>aOR (95% CI)
Age			
	30–39	1.038 (1.008–1.069)*	1.003 (0.953–1.057)
	40–49	0.998 (0.981–1.015)	1.045 (1.009–1.083)*
	50–59	0.989 (0.977–1.002)	0.991 (0.960–1.022)
	60–69	0.988 (0.978–0.998)*	1.011 (0.978–1.045)
	70≤	0.997 (0.989-1.005)	0.993 (0.960–1.026)
Body mass index (kg/m²)			
	0–22	0.995 (0.986–1.004)	1.014 (0.990–1.038)
	23–24	0.995 (0.985–1.004)	0.997 (0.967–1.029)
	25≤	0.992 (0.983–1.001)	0.998 (0.973–1.023)
Income group			
	Low	0.996 (0.985–1.006)	1.009 (0.979–1.039)
	Mid-Low	0.996 (0.986–1.007)	1.009 (0.978–1.041)
	Mid-High	0.997 (0.985–1.008)	1.016 (0.986–1.047)
	High	0.990 (0.979–1.000)*	0.994 (0.958–1.032)
Employment status			
	Employed	0.994 (0.987–1.000)	1.000 (0.979–1.022)
	Unmployed	0.996 (0.987–1.004)	1.006 (0.985–1.028)
Marital status			
	Ever married	0.993 (0.988–0.999)*	1.004 (0.989–1.019)
	Never married	1.008 (0.977–1.040)	0.990 (0.908–1.079)
Alcohol consumption			
	0–4 drinks/sitting	0.993 (0.987–1.000)*	1.008 (0.991–1.025)
	≥5 drinks/sitting	0.994 (0.985–1.003)	1.000 (0.969–1.033)
Aerobic activity			
	No	0.990 (0.983-0.997)*	1.000 (0.982–1.018)
	Yes	1.000 (0.991-1.008)	1.015 (0.988–1.042)
Hypertension			
	No	0.993 (0.986–1.000)*	1.010 (0.993–1.028)
	Yes	0.995 (0.987–1.004)	0.994 (0.965–1.024)

aOR, adjusted odds ratio; CI, confidence interval.
** $p<0.05$*
